# Impact of the pentose phosphate pathway on metabolism and pathogenesis of *Staphylococcus aureus*

**DOI:** 10.1371/journal.ppat.1011531

**Published:** 2023-07-13

**Authors:** Jisun Kim, Gyu-Lee Kim, Javiera Norambuena, Jeffrey M. Boyd, Dane Parker

**Affiliations:** 1 Department of Pathology, Immunology and Laboratory Medicine, Center for Immunity and Inflammation, Rutgers New Jersey Medical School, Newark, New Jersey, United States of America; 2 Department of Biochemistry and Microbiology, Rutgers, The State University of New Jersey, New Brunswick, New Jersey, United States of America; Lunds universitet Medicinska fakulteten, SWEDEN

## Abstract

*Staphylococcus aureus* is an important pathogen that leads to significant disease through multiple routes of infection. We recently published a transposon sequencing (Tn-seq) screen in a mouse acute pneumonia model and identified a hypothetical gene (SAUSA300_1902, *pgl*) with similarity to a lactonase of *Escherichia coli* involved in the pentose phosphate pathway (PPP) that was conditionally essential. Limited studies have investigated the role of the PPP in physiology and pathogenesis of *S*. *aureus*. We show here that mutation of *pgl* significantly impacts ATP levels and respiration. RNA-seq analysis of the *pgl* mutant and parent strains identified compensatory changes in gene expression for glucose and gluconate as well as reductions in the pyrimidine biosynthesis locus. These differences were also evident through unbiased metabolomics studies and ^13^C labeling experiments that showed mutation of *pgl* led to reductions in pyrimidine metabolism including decreases in ribose-5P, UMP and GMP. These nucleotide reductions impacted the amount of extracellular DNA in biofilms and reduced biofilm formation. Mutation also limited the capacity of the strain to resist oxidant damage induced by hydrogen peroxide and paraquat and subsequent intracellular survival inside macrophages. Changes in wall teichoic acid impacted susceptibility to hydrogen peroxide. We demonstrated the importance of these changes on virulence in three different models of infection, covering respiratory, skin and septicemia, demonstrating the need for proper PPP function in all models. This work demonstrates the multifaceted role metabolism can play in multiple aspects of *S*. *aureus* pathogenesis.

## Author summary

*Staphylococcus aureus* is an important cause of a variety of diseases. Due to its propensity to be resistant to antibiotics and the absence of a vaccine, studies to understand its fundamental physiology are important identify potential new avenues for treatment. In this study we investigated the role of one aspect of *S*. *aureus* metabolism: the pentose phosphate pathway. We demonstrated that decreased carbon flux through this pathway not only led to disruptions in the ability of the bacteria to generate energy but also influence multiple facets of its ability to cause infection. We confirmed the impact of these changes in multiple models of infection to reflect the varied diseases *S*. *aureus* can cause. This work demonstrated that metabolism does not merely impact the energy requirements of bacteria but can impact important functions that play direct roles in the organism’s ability to cause disease.

## Introduction

Central metabolism [glycolysis, the pentose phosphate pathway (PPP) and tricarboxylic acid cycle] supplies the biosynthetic intermediates to produce all biomolecules. Bacteria must be able to acquire sufficient nutrients from the environment in order to persist, as well as transmit between hosts. The PPP is a major pathway for glucose metabolism along with glycolysis. While its contribution to metabolism is known [1], the role(s) it can play in metabolic adaption and pathogenesis are not well explored.

*Staphylococcus aureus* is a major human pathogen that leads to significant morbidity and mortality, causing an array of infections from skin and soft tissue, to pneumonia and systemic infection [2]. Methicillin-resistant *S*. *aureus* (MRSA) is prevalent, and an effective vaccine is yet to be developed. In the United States, more people die from MRSA than HIV/AIDS and tuberculosis combined [3]. An understanding of the interaction between *S*. *aureus* and the host may identify new avenues for therapeutic development. One aspect of *S*. *aureus* pathogenesis relates to its capacity to obtain nutrients from the host and thus contribute to its metabolism [4]. We recently conducted a Tn-seq screen in *S*. *aureus* that demonstrated growth and stress tolerance were two independent metabolic strategies used by this pathogen to survive in the airway [5]. One of the genes identified in this screen as being attenuated in vivo was predicted to encode an enzyme within the PPP. Interruption of metabolism in *S*. *aureus* is known to impact virulence [6–11] however, studies on the PPP are few.

In this study we sought to define the impact of the pentose phosphate metabolic pathway on the metabolism and pathogenesis of *S*. *aureus*. Using functional, transcriptional, and metabolomics analyses, we demonstrate that interference with the PPP impacts energy and nucleotide output. This has direct consequences on important functions such as biofilm formation and resistance to host stressors. We show that this impacts virulence using three in vivo major models of infection. This study demonstrates the intimate link that metabolic pathways can have on multiple aspects of pathogenesis.

## Results

### Identification of a pentose phosphate pathway gene and its impact on energy output

**Fig 1:**
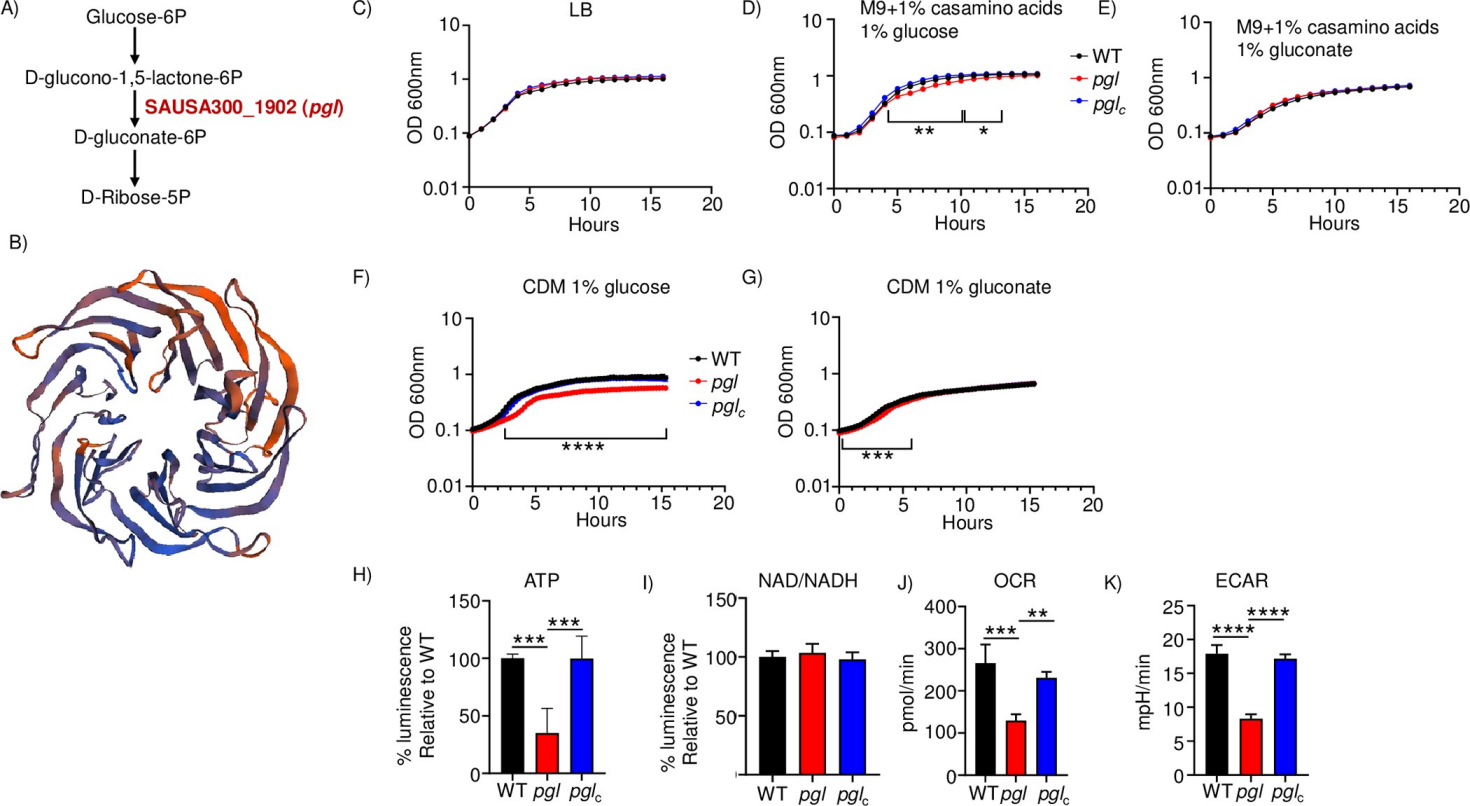
Inactivation of *pgl* leads to reduced energy output. A) Pentose phosphate pathway. B) Model of PGL generated using SWISS-MODEL to a putative phosphogluconolactonase from *Lactobacillus plantarum*. C) Growth in rich (LB), D) M9 media with glucose, E) M9 media with gluconate, n = 3 from representative experiments. Growth in chemically defined media (CDM) with F) glucose or G) gluconate as the carbon source, n = 5. H) ATP production. n = 5. I) NAD/NADH production. n = 4. Respiratory output was quantified using Seahorse analysis. J) Oxygen consumption rate and K) extracellular acidification rates were measured. n = 4. ****P<0.0001, ***P<0.001, **P<0.01.

We recently conducted a Tn-seq screen to identify genes important for pathogenesis in our mouse model of *S*. *aureus* acute pneumonia [5]. One of the genes identified in this screen as conditionally essential in vivo was SAUSA300_1902 (FPR3757 background) which was annotated as a hypothetical gene. To gain more insight into its function we undertook bioinformatic analysis of its sequence. It encodes a 342 amino acid protein (38.5kDa) with 31.69% sequence identity (50% similarity) to 6-phosphogluconolactonase from *Escherichia coli*, encoded by the gene *pgl*. PGL acts within the pentose phosphate pathway (PPP) to convert D-glucono-1,5-lactone-6 phosphate to D-gluconate-6 phosphate (**Fig 1A**). Our analysis indicates that the gene is conserved across staphylococci. It is predicted to be located in the cytoplasm. Consistent with its sequence similarity to PGL we were able to model the predicted *S*. *aureus* protein with PGL from *Lactobacillus plantarum* with a GMQE [12] score of 0.72, meaning we could reliably follow the quaternary structure (**Fig 1B**). It shows a structure bound by beta sheets that facilitates a solvent accessible binding site. Limited studies have been conducted on genes associated with the PPP in *S*. *aureus* [8] so we investigated its role in several aspects of physiology and pathogenesis.

Given its predicted role in the PPP were first tested the growth yield and rate for the SAUSA300_1902 (hereafter referred to as *pgl*) strain along with a complemented strain that had the full *pgl* gene integrated onto the chromosome (*pgl*_*c*_). We did not observe any growth yield or rate defect in rich LB media (**Fig 1C**). The *pgl* mutant did have a delay in growth rate using minimal media (M9) with casamino acids and glucose (**Fig 1D**). Indicative of the role of PGL in the PPP, we did not see this delay in growth if we bypassed the mutation by providing gluconate as the carbon source (**Fig 1E**). The defect of the *pgl* mutant was further pronounced when we utilized a chemically defined medium (CDM). A significant delay to exponential phase and growth yield (39%, P<0.0001) was observed with glucose as the carbon source (**Fig 1F**). When gluconate was provided as the carbon source, while there was a slight delay to exponential phase growth, the *pgl* mutant was able to utilize this substrate to achieve growth metrics equivalent to the WT strain (**Fig 1G**).

We next examined the roles of PGL on metabolic output. Quantification of ATP levels in exponential phase cultures saw a 65% decrease in the *pgl* mutant (P<0.001; **Fig 1H**). We did not detect changes in NAD/NADH levels (**Fig 1I**). We examined the respiration rate of strains using Seahorse analysis. The oxygen consumption rate of *pgl* was 52% lower than the WT strain (P<0.001; **Fig 1J**) and the extracellular acidification rate was decreased by 54% (P<0.0001; **Fig 1K**). We further interrogated the capacity of the *pgl* mutant to utilize different nutritional substrates using phenotype microarrays (Biolog). The *pgl* strain was not able to metabolize the alternative carbon source lactulose (**S1 Table and S1A Fig**). Inactivation of *pgl* also impacted the redox potential and growth ability with different phosphorus sources (**S2 Table and S1B Fig**). Redox potential with cytidine-2 or 3-monophosphate was reduced by 23–30%, likewise with different adenosine monophosphates, suggesting a defect in the ability to metabolize these compounds. Growth in lactulose was confirmed using chemically defined media (**S1C Fig**). These data indicate that *pgl* encodes a predicted protein in the PPP and its mutation leads to significant growth defects, reductions in metabolic output and metabolism of phosphate sources, and subsequent respiration.

### Inactivation of PPP leads to compensatory changes in glucose transport and decreases in pyrimidine biosynthesis

**Fig 2:**
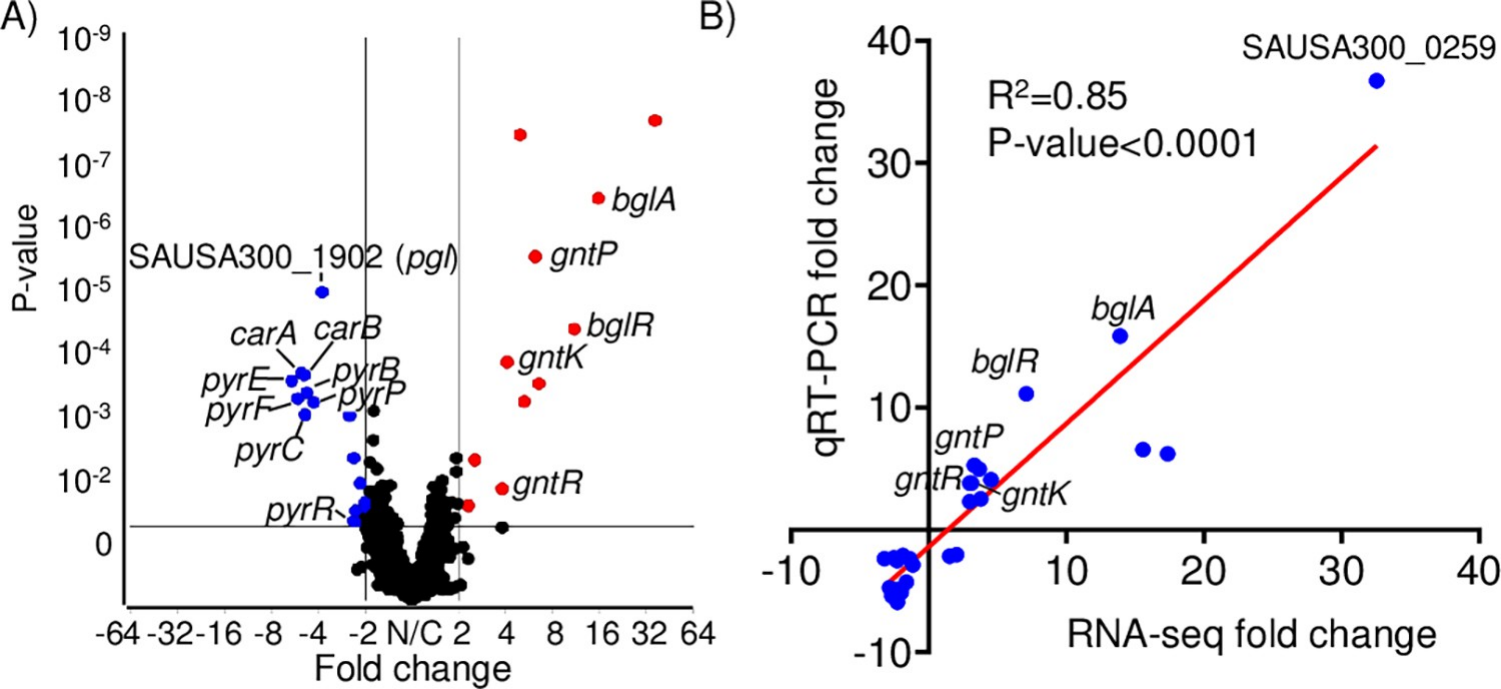
Inactivation of *pgl* impacts pyrimidine biosynthesis. RNA-seq analysis of the mutation in *pgl* compared to WT *S*. *aureus*. A) Volcano plot of differentially expressed genes identified from RNA-seq comparing WT and *pgl* transcriptomes. B) qRT-PCR was conducted on each differentially expressed gene identified from RNA-seq analysis. Correlation plot between independent RNA-seq and qRT-PCR data. n = 6.

To better define the role of PGL in the cell we performed RNA-seq on exponential phase cultures (**Fig 2A**). We identified 28 significant gene changes in the *pgl* mutant (>2-fold, P<0.05; **Table 1**). Differentially expressed genes identified from our RNA-seq analysis were independently confirmed on separate samples by qRT-PCR. We observed a strong correlation between the qRT-PCR and RNA-seq data (R^2^ = 0.85; P<0.0001; **Fig 2B and Table 1**). The observed changes were also validated by qRT-PCR using our complemented strain that indicated the differences were due to inactivation of *pgl* and not secondary mutations (**S3 Table**). The first major observation we observed was compensatory increases in glucose and gluconate production and import. *bglA* which encodes a 6-phospho-β-glucosidase that hydrolyses β-1,4-linked cellobiose 6-phosphate (cellobiose-6′P) to yield glucose and glucose 6-phosphate [13], its regulator *bglR* [14] and a gene encoding a phosphotransferase (PTS) β-glucoside transport protein (SAUSA300_0259) were all significantly increased (**Table 1**). This acts to compensate at the start of the PPP by providing additional glucose (**Fig 1A**). We also observed a significant increase in the *gnt* operon (*gntP*, *gntK*, *gntR*) that transports and converts gluconate to 6-phospho-D-gluconate, acting to increase the defect left by PGL in not converting 6-phoshogluconolactone to 6-phosphogluconate (**Fig 1A**). Inactivation of these genes in *S*. *aureus* did not have a major impact on virulence (**S2 Fig**). The major group of down-regulated genes identified were the *pyr* genes involved in pyrimidine biosynthesis. We predict this is an adaptive mechanism by the cell due to decreased ribose output from the PPP in the absence of PGL.

**Table 1:** Differentially expressed genes in *pgl* vs WT.

		RNA-seq		qRT-PCR	
Gene	Gene ID-putative function	Fold-change	P-value	Fold-change	P-value
	SAUSA300_0259 –PTS system, IIA component	36.735	2.046E-09	32.55061	0.0011
*bglA*	SAUSA300_0260–6-phospho-beta-glucosidase	15.855	5.3193E-08	13.90831	0.0001
*bglR*	SAUSA300_0258 –transcriptional regulator, GntR family	11.124	1.2244E-05	7.099261	0.0001
*scrA*	SAUSA300_2324 –protein = PTS system, sucrose-specific IIBC component	6.5687	0.00011885	15.57982	<0.0001
	SAUSA300_0261 –conserved hypothetical protein	6.2234	6.1317E-07	17.36554	0.0003
*gntP*	SAUSA300_2442 –gluconate permease	5.2868	0.00025352	3.323694	0.0027
*pdxT*	SAUSA300_0505 –glutamine amidotransferase	4.9615	4.5885E-09	3.684173	<0.0001
*pdxS*	SAUSA300_0504 –pyridoxine biosynthesis protein	4.1015	5.2554E-05	4.536557	0.0002
*gntR*	SAUSA300_2444 –gluconate operon transcriptional repressor	3.814	0.04981606	2.999106	0.0027
*gntK*	SAUSA300_2443 –gluconate kinase	3.8128	0.00978853	3.112866	0.0059
*ptsG*	SAUSA300_0191 –PTS system, glucose-specific IIBC component	2.5444	0.00299481	3.796205	0.0009
*glcB*	SAUSA300_2476 –phosphotransferase system	2.3152	0.02007086	2.988264	0.0003
*sstC*	SAUSA300_0720 –putative iron compound ABC transporter	-2.0241	0.02110039	2.041796	0.1462
	SAUSA300_1099 –conserved hypothetical protein	-2.0801	0.03793103	-1.869837	0.0885
*sstB*	SAUSA300_0719 –iron compound ABC transporter, permease protein	-2.152	0.0084108	1.51949	0.2920
*deoD*	SAUSA300_0138 –purine nucleoside phosphorylase	-2.3005	0.02591365	-2.540584	0.0311
*pyrR*	SAUSA300_1091 –PyrR bifunctional protein	-2.3543	0.03900896	-1.387027	0.0223
*tet38*	SAUSA300_0139 –putative tetracycline resistance protein	-2.3613	0.00300309	-3.209246	0.0094
*pyrD*	SAUSA300_2526 –dihydroorotate dehydrogenase	-2.5145	0.00051698	-2.291344	0.0072
*sstA*	SAUSA300_0718 –iron compound ABC transporter, permease	-2.8611	0.00029593	-1.146227	0.4810
*pgl*	SAUSA300_1902 –conserved hypothetical protein	-3.772	3.2143E-06	-3.299748	0.0063
*pyrP*	SAUSA300_1092 –uracil permease	-4.2635	0.00028026	-1.615762	0.0359
*pyrB*	SAUSA300_1093 –aspartate carbamoyltransferase	-4.7399	0.0001934	-2.845135	<0.0001
*pyrC*	SAUSA300_1094 –dihydroorotase	-4.8678	0.00047335	-2.276059	0.0092
*carB*	SAUSA300_1096 –carbamoyl-phosphate synthase	-4.9226	9.2375E-05	-1.997249	0.0067
*carA*	SAUSA300_1095 –carbamoyl-phosphate synthase	-5.1135	8.3923E-05	-2.002157	0.0347
*pyrF*	SAUSA300_1097 –orotidine 5’-phosphate decarboxylase	-5.4067	0.00024603	-2.662519	0.0047
*pyrE*	SAUSA300_1098 –orotate phosphoribosyltransferase	-5.9255	0.00011739	-2.262644	0.0042

### Mutation of the PPP impacts nucleotide production

**Fig 3:**
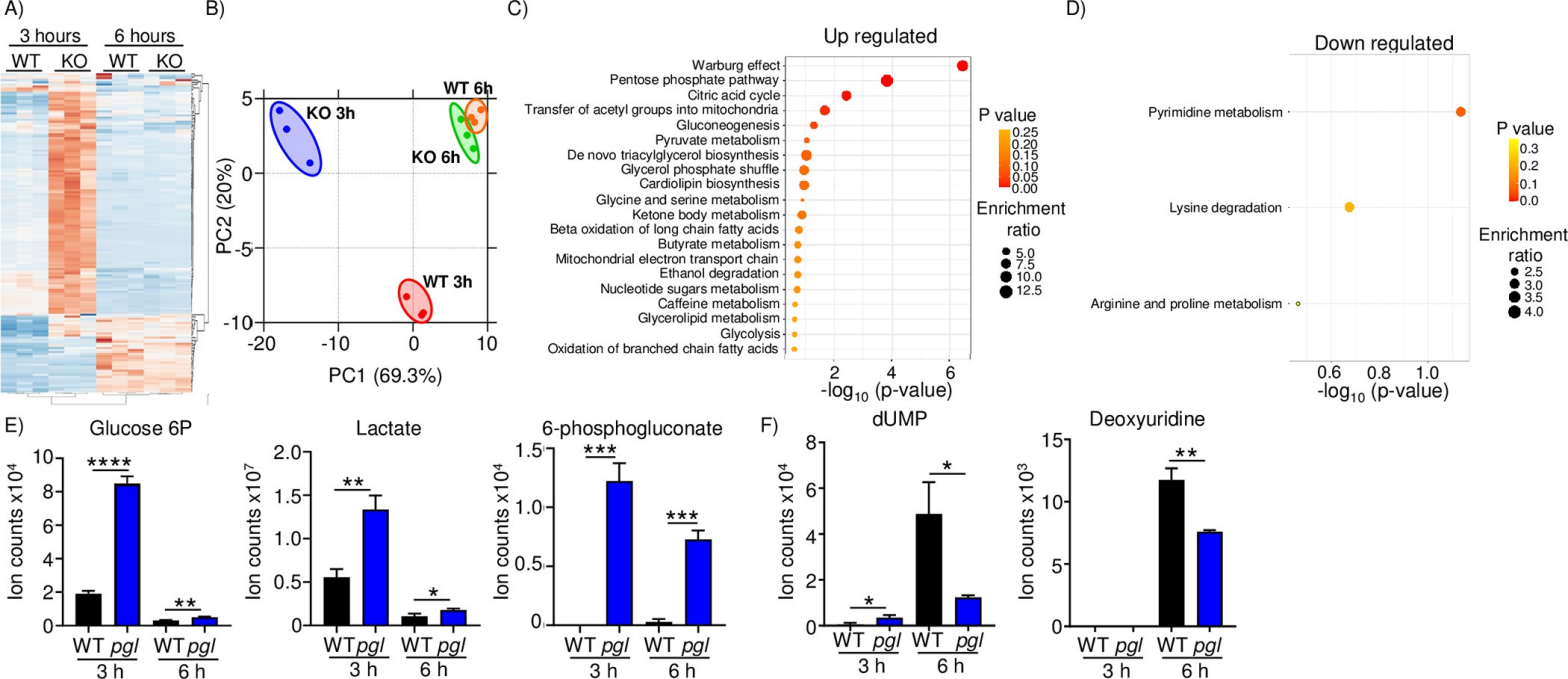
Metabolomic analysis of *pgl* indicates compensatory changes and decreased pyrimidine metabolism. Strains of *S*. *aureus* USA300 were grown in LB for 3 or 6 hours before metabolites were extracted. A) Heatmap of metabolites collected and B) PCA analysis of metabolite production. Metabolite differences (>1.5-fold and p-value <0.05) were analyzed for enrichment of metabolic pathways. C) Up regulated metabolite pathway analysis. Top 15 are shown. D) Down regulated metabolite pathway analysis. E) Individual up regulated metabolites. F) Individual down regulated metabolites. Graphs display means and standard deviations. n = 3. ****P<0.0001, ***P<0.001, **P<0.01 and *P<0.05.

To better understand the physiological impact PGL/PPP plays in *S*. *aureus*, we performed an unbiased metabolomics study after 3 (OD600nm = 1.7, exponential phase) and 6 (OD600nm = 4.3, stationary phase) hours of growth in LB medium. We observed that a large number of metabolites were altered, mostly increased after 3 hours (**Fig 3A and S4 Table**). The number of differentially produced metabolites was reduced by the 6 hour time point (**Fig 3A**). Principal component analysis of these data showed that the wild-type and mutant strains were clustered disparately at the early time point, while they were clustered similarly by the 6 hour time point (**Fig 3B**). Enrichment analysis at this later time point of the increased metabolites in *pgl* indicated a strong and significant presence of metabolites associated with the Warburg effect and the PPP (**Fig 3C**). There were much fewer metabolites that were decreased in the *pgl* mutant by 6 hours, but within this group we saw a significant enrichment for products associated with pyrimidine metabolism (**Fig 3D**). Consistent with our RNA-seq data, we saw significant increases in glucose-6P and 6-phosphogluconate, and further consistent with the Warburg effect and our respiration data (**Fig 1J**), lactate (**Fig 3E**). Amongst the decreased metabolites, we saw a 75% decrease in the amount of dUMP (P<0.05; **Fig 3F**). In a more restricted medium, M9, differences were seen across multiple pathways (**S5 Table**).

**Fig 4:**
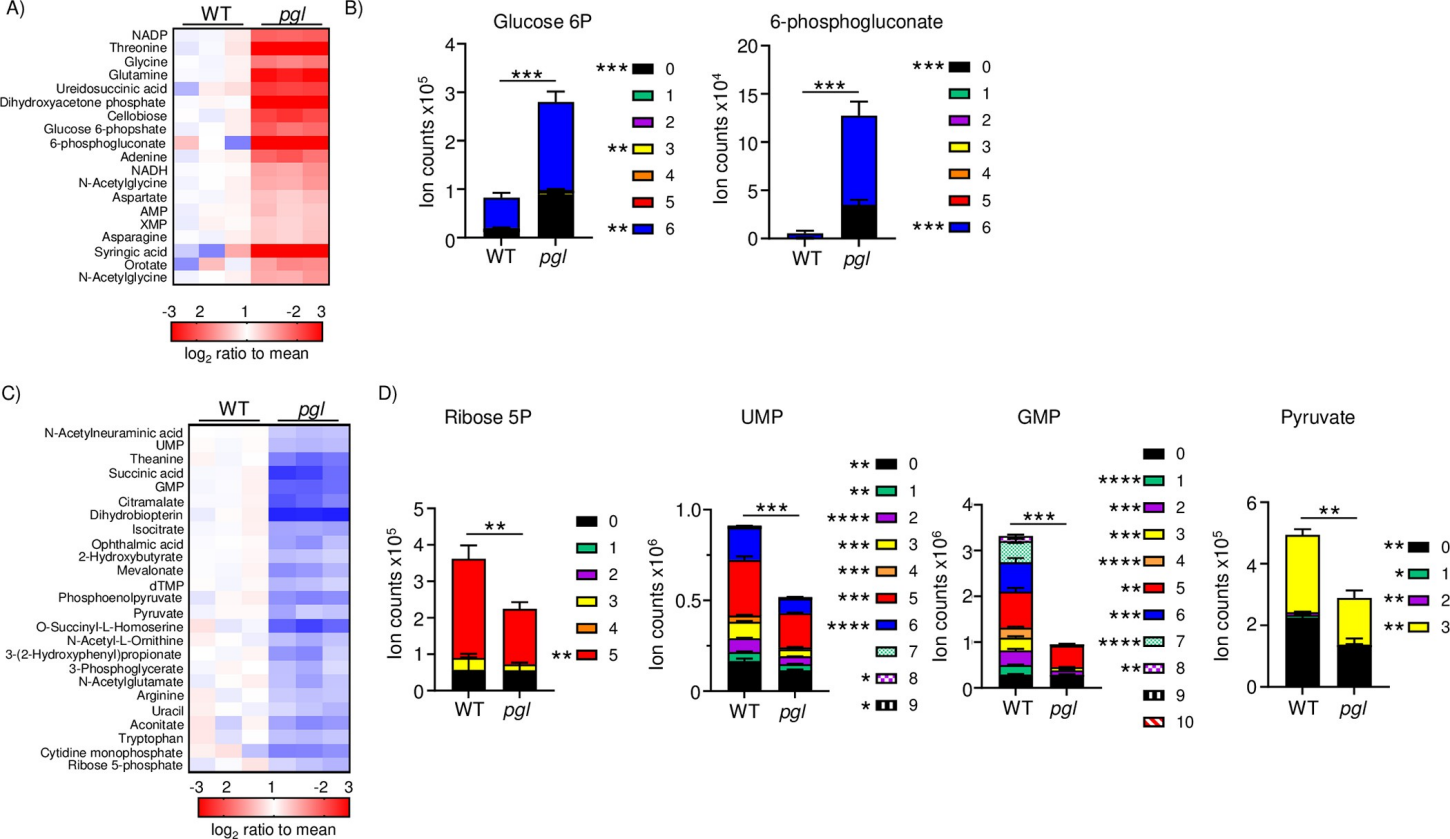
Mutation of *pgl* reduces nucleotide output. *S*. *aureus* was grown to exponential phase before media was replaced with ^13^C_6_ labelled glucose and grown for a further 60 min prior to metabolite extraction. A) Heatmap of up-regulated metabolites and B) selected increased metabolites. C) Heatmap of down-regulated metabolites and D) selected decreased metabolites and their carbon labelling breakdown. Changes >1.-5fold and P<0.05 are shown. Graphs display means and standard deviations of carbon labelled sum. n = 3. ****P<0.0001, ***P<0.001, **P<0.01 and *P<0.05.

We further investigated these metabolomic differences by culturing cells with ^13^C labeled glucose prior to our metabolomic analyses. This process allows us to both understand metabolite abundance but also overall pathway activity. We utilized TSB medium lacking dextrose to facilitate the use of labelled glucose. Under the conditions used, we saw significant differences in the *pgl* strain compared to WT, with both increases and decreases in total (all carbon labels) metabolite levels, as well as labeling in the different carbon positions (**Fig 4A and 4C and S6 Table**). Amongst the increases we again noted the significantly higher levels of glucose-6P and 6-phosphogluconate, at 3 and 24-fold higher respectively in the absence of *pgl* (P<0.001) (**Fig 4B**). We also observed ribose-5P to be significantly reduced in *pgl* (**Fig 4D**). Consistent with the reduction in ribose, amongst the decreased metabolites, we again saw UMP as well as GMP significantly reduced, not just in total but at various states of ^13^C incorporation (**Fig 4D**). We also observed a significant reduction in pyruvate (**Fig 4D**), which we presume is a consequence of reduced ribose-5P being fed into the glycolytic pathway. We did not observe any differences in ribulose. This would suggest that the enhanced levels of 6-phosphogluconate are unable to be converted any faster or PGL is involved in the conversion of 6-phosphogluconate to ribose-5P in the PPP. We observed decreased pathway activity as evidenced by the decreases in labelled carbons at each position within the metabolites. These metabolomics studies support our observations from RNA-seq that interruption of the PPP impacts nucleotide production.

### Decreases in extracellular DNA impact formation of biofilms by the PPP

**Fig 5:**
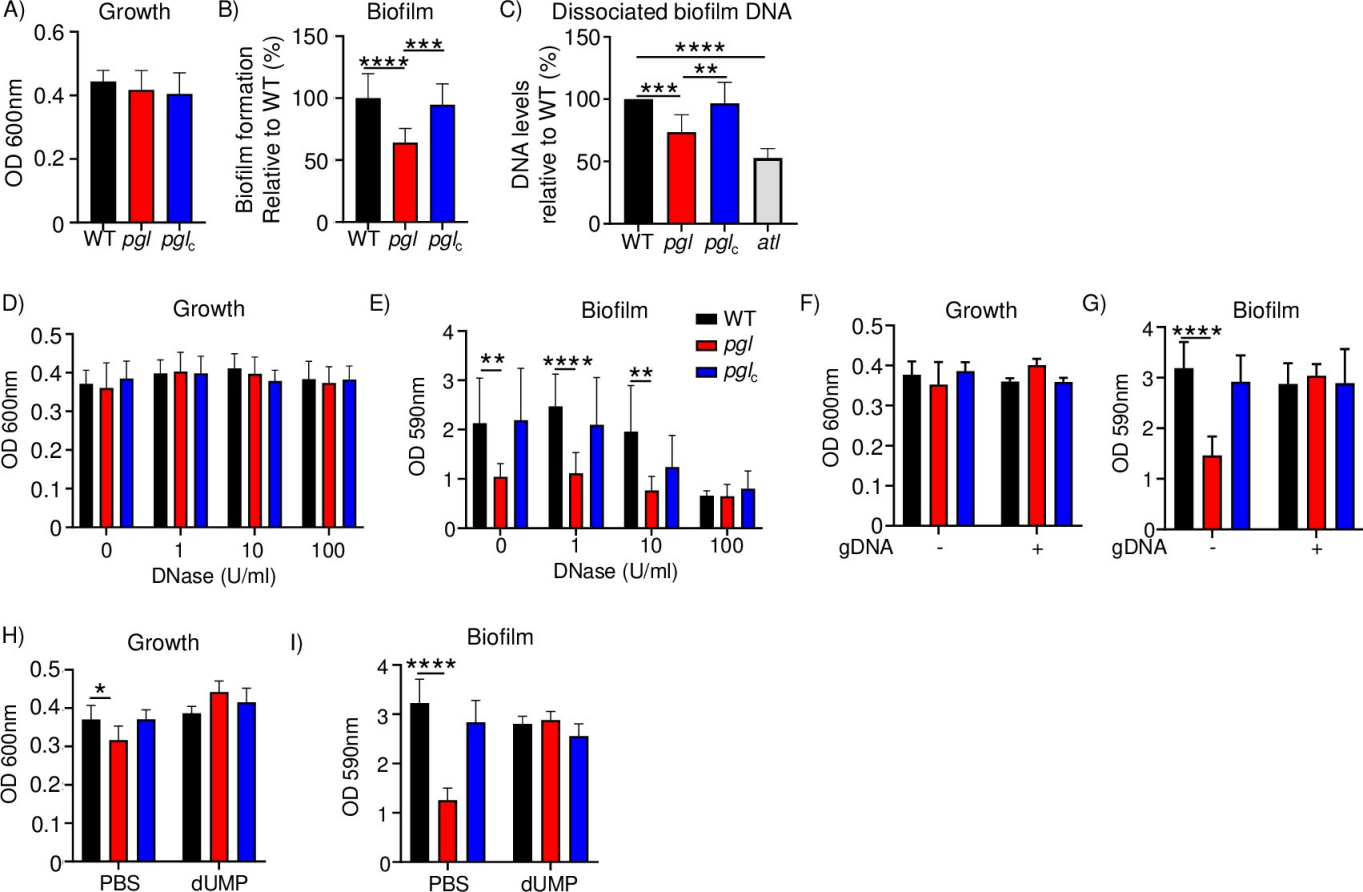
Mutation in *pgl* impacts biofilm and eDNA production. *S*. *aureus* strains were allowed to grow in static cultures for 24 h before A) growth and B) biofilms were quantified. n = 12. C) Biofilms were dissociated, and DNA quantified using Sytox Green dye. n = 8. Strains were grown in the presence of DNase. D) growth and E) biofilm formation. n = 9. Strains were grown in the presence or absence of genomic DNA (gDNA). F) Growth and G) biofilm formation. Without DNA WT and *pgl*_c_ n = 8, *pgl* n = 11.With DNA WT and *pgl*_c_ n = 6 and *pgl* n = 9. H) Growth and I) biofilm in the presence of dUMP. n = 8 without and n = 9 with dUMP. Graphs show means with standard deviation. ****P<0.0001, ***P<0.001, **P<0.01.

We sought to determine what pathogenic traits might be influenced by the observed differences in transcriptomics and metabolomics as a result of the inactivation of *pgl*. A theme amongst these two approaches was a reduction in the pyrimidine operon (by RNA-seq) and the concomitant decrease in nucleotides observed in our metabolomics studies. We reasoned one process that utilizes DNA is biofilm formation. Extracellular DNA (eDNA) is used by the bacterium as a major constituent of the biofilm matrix [15,16]. We observed in the conditions required for biofilm formation, there were no detectable growth defects in the *pgl* mutant (**Fig 5A**). Biofilm formation of the *pgl* mutant was reduced by 36% (P<0.0001; **Fig 5B**) and restored to WT levels in the complemented strain. This requirement for biofilm formation was also evident in M9 media, even though the bacteria are unable to grow as much (**S3 Fig**). To quantify the amount of eDNA, we grew biofilms, then dissociated the biofilm matrix and quantified extracellular DNA with Sytox Green. We compared this to a strain lacking the major autolysin, Atl that would release less extraneous materials to assist in forming the biofilm matrix [17]. We observed a 26% reduction (P<0.001) in eDNA in the *pgl* strain (**Fig 5C**). These data indicate that while PGL does not impact growth it has a decreased capacity to form a biofilm that is due, in part, to decreased presence of eDNA.

We further investigated the impact of the PPP on the DNA composition of biofilms. Biofilms are known to contain DNA [15,16] and we first confirmed this in our assays by demonstrating that increasing concentrations of DNase reduce the ability of *S*. *aureus* to form biofilms (**Fig 5D and 5E**). Consistent with the *pgl* strain producing less eDNA, it was not affected by the DNase, compared to the WT and complemented strains. We were then able to complement the biofilm defect with *pgl* through the addition of sheared *S*. *aureus* USA300 genomic DNA. This was able to restore the *pgl* biofilm to WT levels. Addition of genomic DNA to the WT and complemented strains did not increase their capacity to form biofilms (**Fig 5F and 5G**). We observed that the pyrimidine dUMP was significantly reduced in the *pgl* strain with our metabolomics studies. The addition of this compound was also able to restore the biofilm of *pgl* to WT levels, while again not altering biofilm formation with the WT strains (**Fig 5H and 5I**). This work demonstrates that DNA is important for biofilm formation with *S*. *aureus* and that the defect with *pgl* is due to reduced extracellular DNA from the decrease in pyrimidines driven by the perturbation in the PPP.

### Perturbation of the PPP attenuates oxidant protection and immune cell killing

**Fig 6:**
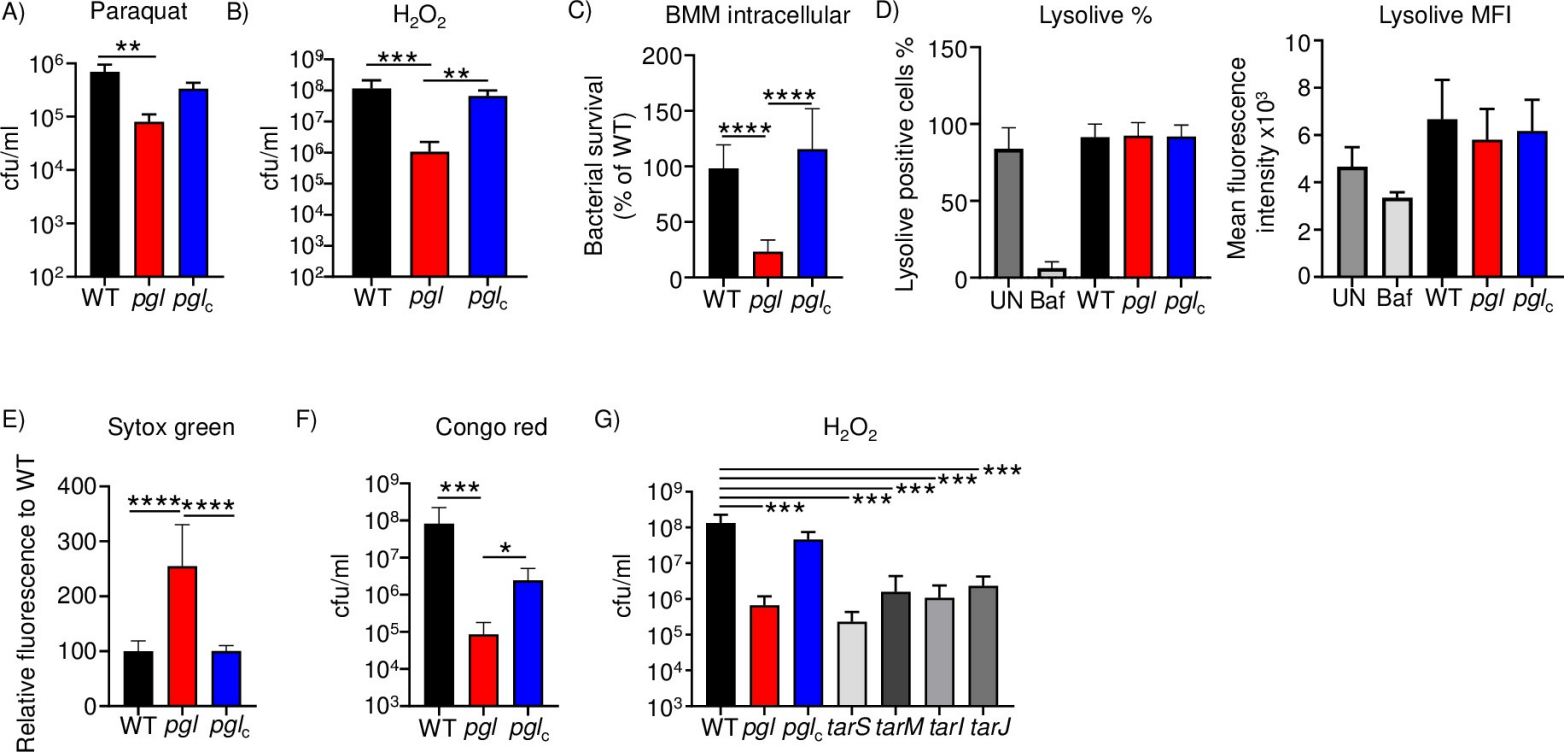
Pentose phosphate pathway aids in protection to oxygen radicals and cellular killing. A&B) *S*. *aureus* strains were exposed to the indicated chemicals for 4 h before bacteria were enumerated. C) Intracellular survival of strains in macrophages. D) Lysolive assay on macrophages incubated with indicated strains. UN-uninfected, Baf-bafilomycin control. Graphs show means with standard deviation. n = 4 for all except H_2_O_2_, n = 10. E) Strains were incubated with sytox green before quantifying fluorescence relative to WT. n = 8. F) Congo red viability assays. n = 8. Strains were grown in hydrogen peroxide for 4 h before quantifying bacteria. G) Controls and *tar* mutant strains. n = 4. Graphs show means with standard deviation. ****P<0.0001, ***P<0.001, **P<0.01.

A major mechanism of killing by professional phagocytes is via reactive oxygen species (ROS). We aimed to test the hypothesis that the decreased metabolic output (reduced ATP) of the *pgl* mutant would make it more susceptible to killing by substances that induce the production of ROS and subsequent bacterial cell death. We first incubated our series of strains with paraquat that generates oxygen radicals within the cell. Within four hours, paraquat led to an 89% decrease in viable bacteria (P<0.001; **Fig 6A**). Treatment with hydrogen peroxide also led to a 47% decrease in viability of the *pgl* mutant compared to the WT strain (P<0.05; **Fig 6B**). These observations translated to decreased intracellular survival in macrophages. The *pgl* strain exhibited a 77% decrease (P<0.0001; **Fig 6C**) in intracellular macrophage survival. This difference may have been the result of differential intracellular pH induced by the different strains. However, measurement of the internal acidity of the macrophages using the dye lysolive indicated this was not the case, compared to a bafilomycin control that reduces intracellular pH (**Fig 6D**). These data show that lack of PGL and PPP function attenuates *S*. *aureus* survival to macrophages and their microbicidal products.

We further investigated the mechanism behind the increased susceptibility of the *pgl* strain to hydrogen peroxide. To address this question, we considered how *S*. *aureus* protects itself from oxygen radicals. Our initial hypothesis was a reduction in catalase, and we show that a catalase mutant is unable to survive treatment with hydrogen peroxide (**S4A Fig**). However, examining the response of the PGL mutant to WT and complemented strains, we did not observe a difference in catalase gene expression, or oxidative stress genes by qRT-PCR under similar assay conditions (**S4B Fig**). Hydrogen peroxide and the subsequent hydroxyl radicals are known to damage the cell wall [18,19]. We thus hypothesized that the *pgl* strain may have changes to its cell wall influencing its susceptibility to hydrogen peroxide. To quantify changes in permeability we incubated our strains with the dye sytox green. We observed that the *pgl* strain permeabilized 150% more dye (P<0.0001; **Fig 6E**) than the WT or the complemented strain. A major component of the cell wall are teichoic acids. We demonstrate that in the absence of PGL, *S*. *aureus* was attenuated in its capacity to grow in the presence of congo red, which targets wall teichoic acids (WTA) [20] (120-fold reduction; P<0.001; **Fig 6F**). WTA are important for colonization and their mutation leads to increased sensitivity to temperature [21–23]. WTA biosynthesis is mediated by enzymes encoded by the *tar* (teichoic acid ribitol) operon [24]. The role of WTA in our hydrogen peroxide killing assay was confirmed with four different teichoic acids mutants (*tarS*, *tarM*, *tarI*, *tarJ*) that exist in the *S*. *aureus* Nebraska Transposon Mutant Library [25]. All four *tar* mutant strains exhibited significant (P<0.001) attenuation compared to the WT control (**Fig 6G**). TarM attaches N-acetyl-D-glucosamine (GlcNAc) in the α configuration at the C4 hydroxyl to ribitol-5-phosphate and TarS attaches GlcNAc in the β configuration at same position as TarM. TarI and TarJ synthesize the ribitol-5-phosphate backbone [24]. TarO aids in the synthesis of the conserved linkage unit of WTA [24]. These data suggest that WTA are influenced by the PPP and their dysregulation impacts susceptibility to oxidant stress.

### Mutation of the PPP impacts pathogenesis across multiple sites of infection

**Fig 7:**
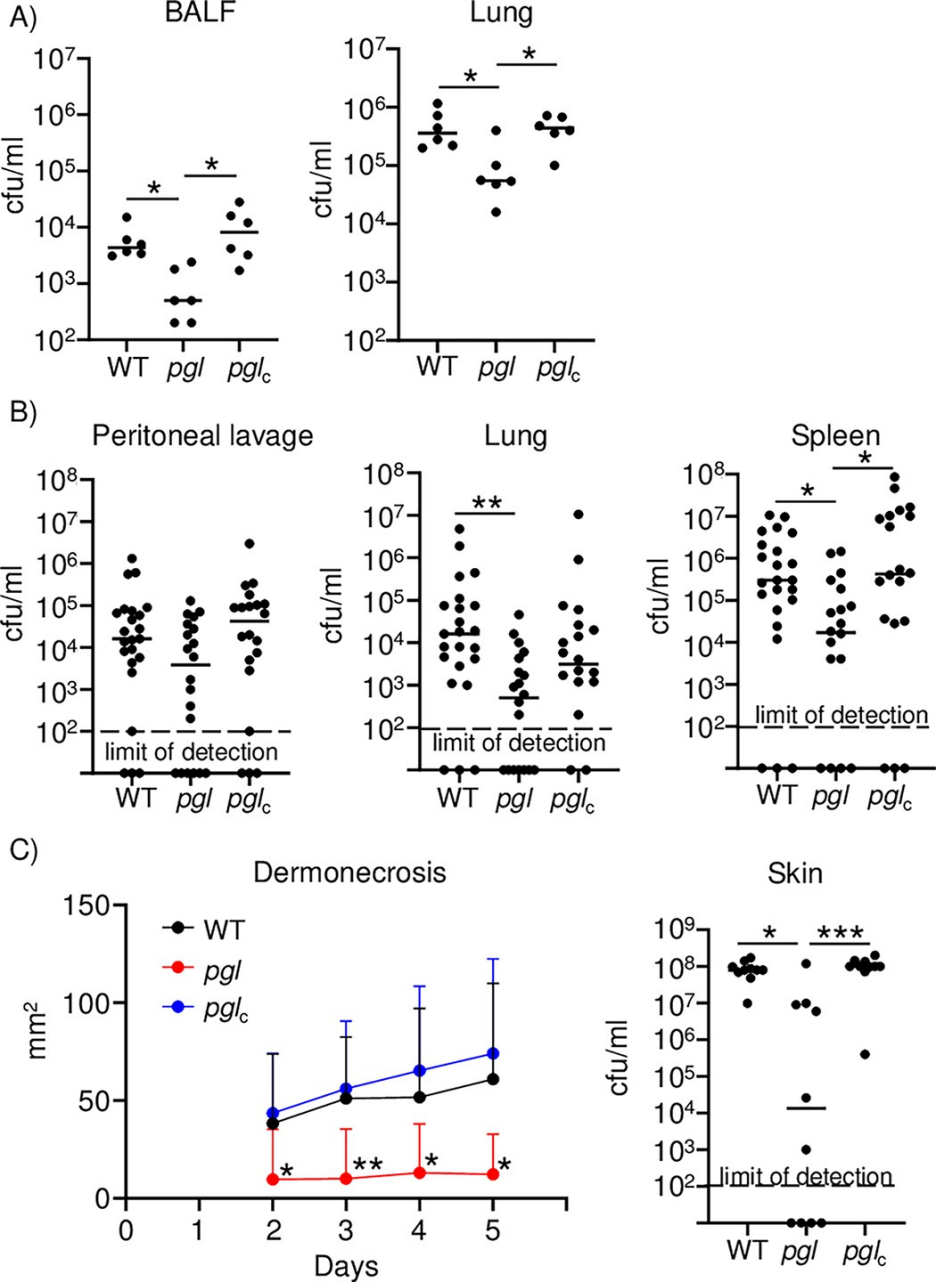
Interruption of the pentose phosphate pathway attenuates *S*. *aureus* pathogenesis. Strains of *S*. *aureus* USA300 were inoculated into mice to assess pathogenicity. A) Model of acute pneumonia using intranasal infection, n = 6. B) Sepsis model using intraperitoneal inoculation. n = 23 for WT and 20 for others. A&B) Bacterial burdens were quantified 24 h after infection. C) Skin and soft tissue infection model. Mice were infected subcutaneously and followed for 5 days. n = 10. Each point represents a mouse. Lines display median. Dermonecrosis charts mean and error. ****P<0.0001, ***P<0.001, **P<0.01.

To define the impact of the physiological changes observed in the absence of *pgl* we tested the ability of *S*. *aureus* to infect mice in its absence across multiple routes of infection. We tested its role in a model of acute pneumonia first, since it was initially identified in our Tn-seq screen in the lung [5]. Intranasal infection with *pgl* led to an 85% decrease in bacterial numbers (9.3 x 10^2^ vs 6.0 x 10^3^ cfu/ml; P<0.05) by 24 hours in bronchoalveolar lavage fluid (BALF) (**Fig 7A**). This attenuation was not evident with the complemented strain, which was as virulent as the parent strain. Consistent with the BALF data, we also observed significant decreases in homogenized lung material (**Fig 7A**).

We next interrogated the capacity of the *pgl* mutant in a model of septicemia. Mice were infected with *S*. *aureus* intraperitoneally and then examined 24 hours later. While we observed on average a 84% decrease in bacterial burden in the peritoneal lavage, this was not statistically significant, likely due to the variation between experiments and animals (**Fig 7B**). We did, however, observe a significant decrease in bacterial spread to the lung in the absence of *pgl*, 99% (4.5 x 10^3^ vs 3.5 x 10^5^ cfu/ml; P<0.01; **Fig 7B**). Consistent with this decrease in dissemination, we also observed that there was an 89% decrease in bacterial spread to the spleen in the absence of *pgl* (2.0 x 10^5^ vs 1.8 x 10^6^ cfu/ml; P<0.05; **Fig 7B**).

Lastly, we determined the impact of mutation of *pgl* over the course of several days in a model of skin and soft tissue infection via the subcutaneous route. In the wild-type and complemented strains we observed consistent regions of dermonecrosis evident from day two, with a trend to increasing size as time went on (**Fig 7C**). In the absence of *pgl*, regions of dermonecrosis were significantly reduced, so much so that 6/10 mice did not develop any noticeable signs of infection (**Fig 7C**). At day five, the average region of dermonecrosis was 61 mm^2^ in wild-type and 12 mm^2^ in the *pgl* mutant (P<0.05). At the conclusion of the experiment, bacterial burdens in mice were consistent with the dermonecrosis data. Median numbers in the *pgl* strains were several orders of magnitude lower, and the average number of *S*. *aureus* was 84% lower (1.5 x 10^7^ vs 8.7 x 10^7^ cfu/ml; P<0.05; **Fig 7C**). These data show that while *pgl* did not have a major impact on growth when cultured on solid or liquid media, the metabolic differences and subsequent impact on pathogenic traits led to significant attenuation in primary models of *S*. *aureus* infection.

## Discussion

In this study, we investigated the function of a gene identified from our prior Tn-seq screen that was conducted in an acute pneumonia model of *S*. *aureus* infection. Microbiological, bioinformatic, biochemical and metabolomic studies confirmed the predicted function of the gene product in the PPP. These studies identified that an additional defect of the mutant strain was decreased production of pyrimidines as observed at both the RNA level and through metabolomics. We demonstrated that these observed changes impacted the persistence of the organism in biofilm formation, extracellular DNA production and resistance to innate stressors and professional phagocytes. We also were able to show the defect of this strain in not only pulmonary infection but systemic and skin and soft tissue models.

We observed an increase in 6-phosphogluconolactone which would not be expected based on the predicted role of PGL in the pentose phosphate pathway. We also saw that even in defined media with glucose as a carbon source, the *pgl* strain, although reduced, could still grow to some extent. To explain this, it has been shown in *E*. *coli* and *L*. *monocytogenes* that inactivation of *pgl* can lead to an accumulation of gluconate, which could then be transported back into the cell [26,27]. It has also been shown that accumulating 6-phosphogluconolactone can be dephosphorylated, then exported, where it can subsequently be hydrolyzed to gluconate then taken back up by the cell and thus bypassing the defect in the pentose phosphate pathway [26,27]. With this in mind, it would account for the partial growth still observed in our CDM however, we do still observe a significant defect in growth that can only be restored with additional exogenous gluconate. Complex formation between components of the pentose phosphate pathway has also been observed, thus interaction of PGL with the next enzyme in the pathway is also possible for maximum output [28].

It is now appreciated that metabolism can influence functions beyond the core role of balancing the energy requirements within a bacterial cell. It has been observed in the other central metabolic pathways of *S*. *aureus*, glycolysis and the TCA cycle that these pathways are linked to virulence [4,7,9,29], and that their influence on virulence acts independently of their effects on metabolism [9,30,31]. It has also been observed that metabolic pathways can coordinate expression of virulence factors, sensing changes in the environment that alters both metabolism and virulence [11,32]. This is an observation that is also witnessed clinically with the generation of small colony variants (SCV) of *S*. *aureus*, which have decreased metabolism and are associated with chronic infections [33]. While the *pgl* strain didn’t look like a SCV the overall colony size was slightly smaller. We did not observe any evidence that inactivation of *pgl* within the PPP pathway led to any differential regulation of other transcriptional factors, as evidenced by our RNA-seq data. It is likely that the influence the PPP had on virulence was more directly tied to its metabolic defects. It was interesting to note that the influence the PPP had on pyrimidine production did occur at the RNA level, suggesting the cell has a feedback mechanism for this arm of metabolism. It has been shown by other groups that pyrimidine biosynthesis is important in both murine models of infection [34] as well as influencing the spread of MRSA within the community [35]. This work also forms a good platform for the development new antimicrobials. As mutation of *pgl* led to increased Warburg effect with reductions in pyrimidine metabolism including decreases in ribose-5P, UMP and GMP. Here, the effect on the formation of UTP in the bacteria leads to the fall in the peptidioglycan biosynthesis and RNA biosynthesis thereby weakens the bacteria and kills *S*. *aureus* that has been seen in other studies interfering with pyrimidine metabolism [36–39].

Until now, little was known about the role PPP in the pathogenesis of *S*. *aureus*. To date, only one other study had sought to determine the impact of the PPP, by studying the transketolase. The transketolase was found to be important for colonizing the kidneys and influencing regulation of other regulatory molecules [8]. Other than this instance and our own data no other mutants have been identified nor able to be generated highlighting the importance of this pathway [5,35,40,41]. Some evidence to the role of the PPP in pathogenesis can be seen in other species also. *Salmonella enterica* serorvar Typhimurium has three transketolases that when all three are lacking it results in the strain being avirulent [42]. While in *Francisella* [43], mutation within the PPP impairs replication within macrophages where it acts as a major metabolic hub, with multiple connections to TCA glycolysis and other fatty acid synthesis.

Central carbon metabolism is an essential process of which the PPP is one element. While the role aspects of these pathways play in metabolism is assumed, our understanding of their precise contributions to metabolism and how they may contribute to pathogenesis is more limited. Investigation of these connections between metabolism and virulence is important as virulence linked pathways are potential therapeutic targets. The data presented here demonstrate that the PPP pathway of *S*. *aureus* not only impacts metabolism but influences multiple aspects of virulence. We show that mutation of the PPP leads to changes in nucleotide synthesis that impacts biofilm formation and interruption of the PPP influences the cell wall to alter oxygen radical sensitivity. These findings all contribute to the attenuation we observed in vivo in several models of infection. This provides new information on both the contribution of the PPP to the metabolic output of *S*. *aureus* but also to aspects of pathogenesis and its importance in survival within the host.

## Methods

### Ethics statement

Animal work in this study was carried out in strict accordance with the recommendations in the Guide for the Care and Use of Laboratory Animals of the NIH (National Academies Press, 2011), the Animal Welfare Act, and US federal law. Protocols were approved by the Institutional Animal Care and Use Committee of Rutgers New Jersey Medical School of Newark, New Jersey, USA.

### Bacterial strains and growth conditions

The *S*. *aureus* strains used in this study were on the JE2 USA300 background. WT, SAUSA300_1902::Tn (*pgl*), *bglA*, *gntK*, *gntP*, *tarS*, *tarM*, *tarI* and *tarJ* from Nebraska Transposon Mutant Library [25] and *pgl* complemented strain were grown at 37°C with 300 rpm shaking in Luria Bertani (LB) medium. SAUSA300_1902::Tn was complemented using the vector pLL39 as before [5]. Growth curves for *S*. *aureus* were conducted in LB, M9 minimal medium (2 mM magnesium chloride, 100 M calcium chloride, 1% casamino acids and 1% glucose or gluconate) or chemically defined media (CDM) [44] with the following modifications: 50 μM FeSO_4_, 1 μM MnCl_2_ and omitting bases (adenine, cytosine, guanine, thymine, uracil) [45] and trace elements (ZnCl_2_, Boric acid, CoCl_2_, CuCl_2_, NiCl_2_, Na_2_MoO_4_) from the original reference [44]. Growth curves were generated from overnight cultures that were diluted 1:100 and optical density of growth at OD_600nm_ was measured for 16 h with 15 min intervals at 37°C using a SpectraMax i3x (Molecular Devices). Strains were grown to OD600 = 1.0 and resuspended in PBS before incubation with 5 μM of sytox green for 2 h at room temperate. Cells were washed three times and fluorescence quantified at excitation of 504 nm and emission at 523 nm (Spectramax i3x). Fluorescence was normalized to bacterial numbers. Congo red assays were conducted by spotting exponential-phase cultures on TS agar plates containing 0.08% (w/v) congo red and enumerating colonies after overnight growth at 37°C. Sensitivity to paraquat and hydrogen peroxide was conducted on exponential-phase bacteria grown in LB (OD600  = 1.0), diluted 1:100 from overnight cultures. *S*. *aureus* was incubated with 100 mM of paraquat or 500 mM of hydrogen peroxide in PBS for 4 h, then serially diluted onto LB agar plates to determine surviving bacteria. The effects of tunicamycin were determined by growth strains to exponential phase in the presence of 2 μg/ml tunicamycin.

### Tissue culture

Bone marrow derived macrophages (BMDM) were generated as described previously [46]. Macrophages were seeded onto 96 well plates at a density of 50,000/well and incubated with *S*. *aureus* at an MOI of 10 for 2 h, then washed and treated with 500 μg/ml of gentamicin for 2 h. Cells were then washed with phosphate-buffered saline (PBS), detached with 100 μl of TrypLE Express (Invitrogen), and serially diluted onto LB agar plates. Lysolive pH-Sensor Green (0.5 μM, Marker Gene Technologies) was added to each well for 1 hour to determine endosomal acidification. Cells were then washed with phosphate-buffered saline (PBS), detached with 100 μl of TrypLE Express (Invitrogen), and analyzed using LSR II flow cytometer (BD Biosciences). Bafilomycin (1 μM) was used as a control.

### Bioinformatics

Basic amino acid sequence predictions utilized Protparam, BLASTP and PSORTb [47–49]. Modelling of PGL was conducted with SWISS-MODEL [50].

### ATP and NAD/NADH quantification

The level of ATP or NAD/NADH was measured using the BacTiter-Glo Microbial Cell Viability Assay (Promega) or NAD/NADH-Glo Assay (Promega) according to manufacturer’s instructions. Diluted overnight cultures in LB were grown to OD 1.0 at 600nm. Cultures were mixed with equal volume of reagent and incubated to detect luminescence.

### Oxygen consumption rate (OCR) and Extracellular acidification (ECAR) measurements

Bacterial basal respiratory activity was quantified using the Seahorse XF HS Mini Analyzer according to the manufacturer’s instructions. *S*. *aureus* was diluted 1:200 from overnight cultures in LB and grown to mid-exponential phase at 37°C. Cultures were diluted to OD_600_ = 0.025 and 180 μL of diluted cells was dispensed into each well of cell culture microplates precoated with poly-D-lysine (glycolysis stress test kit). The seahorse XF sensor cartridge was hydrated in a non-CO_2_ 37°C incubator with sterile water (overnight) and pre-warmed XF calibrant for 60 min prior to measurement. Each bacterial strain was enumerated to confirm equal concentrations and run on the machine. Mixing and measurement time was set to three minutes in each cycle.

### Phenotype Microarrays

Metabolic panels PM1 and PM4 (Biolog) were used in this study. Bacterial strains were cultured overnight in LB and subsequently diluted at 1:100. Exponential-phase growth (OD600 = 1.0) cultures were diluted to achieve 81% transmittance in IF-0a inoculating fluid (Biolog), and then used to prepare the PM plate inoculum as follows. Each PM plates and solutions were purchased from Biolog and prepared according to the manufacturer’s protocol for Gram-positive bacteria. 12x additive solution for PM1 plate included 24 mM MgCl_2_, 12 mM CaCl_2_, 0.3 mM L-arginine, 0.6 mM L-glutamate, 0.15 mM L-cystine (pH8.5), 0.3 mM 5’-UMP, 0.06% (w/v) yeast extract, and 0.06% (v/v) Tween 80. 12x additive solution for PM4 plate contained 240 mM tricarballylic acid (pH7.1), 24 mM MgCl_2_, 12 mM CaCl_2_, 0.3 mM L-arginine, 0.6 mM L-glutamate, 0.06% (w/v) yeast extract, 0.06% (v/v) Tween 80, 30 mM D-glucose, and 15 mM sodium pyruvate. Inoculating fluid for PM panels was assembled by combining 10 ml of IF-0a, 0.12 ml of 100x redox Dye H, 1 ml of 12x PM additive solution and 0.88 ml of 81% transmittance bacterial cells. 100 μl of inoculating fluids were dispensed into each well of PM1 and PM4 plates. Plates were incubated for 24 h at 37°C without shaking. After incubation, optical density of growth in each well was measured at 590 nm by a SpectraMax i3x (Molecular Devices) plate reader.

### Biofilm assay

Bacterial cultures grown in LB were diluted 1:100 from exponential-phase culture (OD600 = 1.0) in Trypticase soy broth (TSB) medium with 0.5% glucose in 96-well tissue culture plates or M9 medium as described above. Static bacterial cultures were grown for 24 h at 37°C, and growth was measured (OD600). Plates were washed twice with water, dried, and stained with 1% crystal violet. After 30 min, plates were washed three times with water and dried before dissolving crystal violet in 100% ethanol with shaking for 15 min and measuring at 590 nm. Growth in the presence of DNase (Roche) was conducted with stated concentrations. Biofilm formation in the presence of DNA (5 ng/μl) was conducted using DNA isolated from exponential phase cultures using the DNeasy kit (Qiagen) according to the manufacturer’s instruction. DNA was then sheared using a Bioruptor Pico (Diagenode), 5 cycles of 30 seconds on then 30 seconds off. Fragmentation was confirmed by agarose gel electrophoresis. A concentration of 10 mM dUMP (VWR) was used in biofilm experiments. Dissociated biofilm was accessed by staining using a SYTOX Green (Invitrogen) reporter dye. Bacterial cells in 96-well tissue culture plates were grown as described above, and the media was removed from each well, and biofilm cells were resuspended with PBS to collect dissociated biofilms. After vigorously pipetting with slight scraping, the resuspended biofilm cells in PBS were pooled and filtered. 100 μl of the filtered resuspension was mixed with 100 μl of 2 μM SYTOX Green in PBS. Fluorescence was measured by using SpectraMax i3x (Molecular Devices) plate reader with excitation and emission wavelengths of 465 nm and 510 nm, respectively.

### RNA-seq

RNA was isolated from exponential phase cultures in LB using the E.Z.N.A RNA kit (Omega Bio-Tek), after 15 min incubation in 50 μM Tris-HCl, 10 μM EDTA pH 8 and 60 μg/ml lysostaphin at 37°C. Isolated RNA was further treated with DNase according to the manufacturer’s instructions (Ambion). RNA was depleted of rRNA (Ribo-Zero; Illumina) before cDNA libraries were constructed and sequenced (Novogene). Sequencing data was analyzed using Partek Flow as before [51]. Data was aligned to the *S*. *aureus* USA300 genome using Bowtie 2 and trimmed mean of M value normalize data with ANOVA and Limma-voom variance. Genes with changes greater than 1.5-fold and p-values less than 0.05 were considered significant. RNA-seq data was deposited in NCBI GEO as GSE136543.

### qRT-PCR

*S*. *aureus* strains were grown in LB medium until an optical density of 1.0 at 600 nm. Total RNA was extracted using the E.Z.N.A RNA kit (Omega Bio-Tek), treated with DNase (Ambion) and cDNA was synthesized using random hexamers and reverse transcriptase (Applied Biosystems). Quantitative reverse transcription PCR was performed using SYBR Green (Applied Biosystems) on a QuantStudio 6 real-time PCR machine.

**Table:** 

**Gene**	**Forward primer**	**Reverse primer**
*carA*	TGGCTCCAGATGGCGTTATG	GCACCACGATGACCAAACTTC
*carB*	CGAGCTTTAGGTATCGAAGGTG	AGCTAACGCTGATGAACGTG
*pyrE*	TGCAACAGCTGGTATTCCAC	ACCTTCACTTTTAGCACCTTCG
*pyrP*	TTACGTCATTGGTGGTGCAG	GCCACTTGCTGCAATAATACCG
*pyrF*	CTGCTGGTGGCGTAAAAATG	GCTGCATTTGCTAACTTGGC
*pyrB*	ATTGCGAATGCTGGTGATGG	TGGTAATTACTACGTGCGACAC
*pyrC*	TGGTGGTGCAATGCATGAAG	ATGAATGCCTGCGCGTTTAG
*pyrR*	TGCTGTATACTGGTCGAACGG	TGTCCTCGATCAACCAAAGC
*pyrD*	AAGTCGAAGAAGGCGGTTTG	AATAAGTGACGCACCGTGAC
*SAUSA300_0259*	AGTAATCGCACCGTTCAATGG	TGCCCTGCTTCAACATGATC
*SAUSA300_0139*	TCAAACTGCCGGTTTAGCTG	ACTGCTCGTACTTAAGCCAAGG
*SAUSA300_0718*	ATGGGTGGGATTGTTGCAAG	GCGTCCACTTGTGATAATGGC
*SAUSA300_0138*	AATTCGCGTTGGCTCTTGTG	GCAATTGGCGCAAAATGACC
*SAUSA300_0719*	GCTTTTAGGTCGTGCTGAAGC	GGTCCTACTAATGCAGTTGACAC
*SAUSA300*_*1099*	GGGAAGATAAAACATTGTAACG	GTTTTTGCTTCGTTGAACTTTCG
*bglA*	ATGTTGGATGGCAAGAAGGG	AACCACCGTTGACGATGTTG
*SAUSA300_0720*	CGTTTTGAGAACGGTGAAGTG	CGTCCAAAGTTTACCAACTGC
*gntK*	ATTCGCGCTGCATTAGAAGG	TTCGCAAAACCACCTGTTGC
*gntR*	TGTGAGTCGTTCGCCAATTC	AAACGGCAACACATGTGCAC
*gntP*	CACGAGAAGGCGATATTTCAGC	ATGCCCAGTTACAAGTTGCG
*SAUSA300*_*0504*	GTGTTAACACCAGCAGATGAGG	AGCAGCACCTTCACCAATTC
*SAUSA300*_*2476*	TAGCCGCATGGTGTTACAAC	AAGCACACCTGTTGCGATTG
*SAUSA300*_*0261*	AGCCTGGGGGTATCTTGTTAAC	AACCAGCCAAGTTTCGTCTG
*SAUSA300*_*0505*	TGGCGAGTCTACAACGTTACG	ATCCTGCGCATGTACCAAAC
*SAUSA300*_*2324*	ACAAGGTGGTGCAGCAATTG	ACACCAAACATAGCCGGTTC
*ptsG*	ACATTTTCCACGCACCGTTC	TGTGCGCCTTCACGAATTTG
*SAUSA300*_*0258*	CGGTGATAAATTGCCAAGCG	ATACCACTGCCTTGTGCTTG
16S	GCGCTGCATTAGCTAGTTGGT	TGGCCGATCACCCTCTCA
*katA*	AATGCATGCCAAAGGTTCG	TGTCACGCTCCGCATCA
*ahpC*	CTCAACGTGGTACATTCATTATCG	TTTAGCGCCTTCTTCCCATTTAG
*sodM*	CGGAGCGATGAGGATGTCA	ATCTAAGTGCCCCACTGCG
*sodA*	AGCGTGTTCCCATACGTCTA	CACGCTTTGGTTCAGGTTGG
*hemQ*	ATCGCGTTCGTCCTTTGGAA	TGAGTCAAGCAGCCGAAACA
*hemG*	TCTCGCAATTACGAAGCCAGT	GGTTCGGACAAGATCCAGCA
*hemN*	ACTCGCCTGTGATTGTAAACG	ACCATGTATGTAGGTGGCGG
*bsaA*	GTGTTGTGCCGCAGTCAAAT	ACCTGGTTCAGGCGAAGAAG
*gpxA2*	TCATCGTTCCCGTTCACAGA	TCGTGGGTTTGTAGTGTTGAGT
*tpx*	ACTGCACGAGCTAATAAGCGA	TGCGCTTCAGCAGGTTTAGA
*quoE*	TCGCCTTTAGCACTACCATCC	TTCGGTCCAACCATTCAGGG
*sarA*	CGTTGTTTGCTTCAGTGATTCG	GTAATGAGCATGATGAAAGAACTGT

### Metabolomics

For metabolomic analyses, overnight *S*. *aureus* cultures were diluted into fresh LB to OD600 nm = 0.1. Five milliliters of culture was grown in 25 mL capacity culture tube for 3 or 6 hours before collecting the cells. For the carbon labelling analysis, cells were grown overnight in TSB and diluted to OD600nm = 0.1 in no dextrose TSB (BD) supplemented with 11 mM glucose (Sigma) and grown for 3 hours. Cells were centrifuged and washed twice with PBS. Cell pellets were suspended in 11mM D-Glucose-^13^C_6_ (389374, Sigma-Aldrich) and incubated for 60 min before collection. Samples for the metabolite profiling were prepared described before [52]. The samples were stored at -80°C until metabolite analysis was performed.

The hydrophilic interaction liquid chromatography (HILC) separation was performed on a Vanquish Horizon UHPLC system (Thermo Fisher Scientific, Waltham, MA) with XBridge BEH Amide column (150 mm × 2.1 mm, 2.5 μm particle size, Waters, Milford, MA) using a gradient of solvent A (95%:5% H_2_O:acetonitrile with 20 mM acetic acid, 40 mM ammonium hydroxide, pH 9.4), and solvent B (20%,80% H_2_O:acetonitrile with 20 mM acetic acid, 40 mM ammonium hydroxide, pH 9.4). The gradient was 0 min, 100% B; 3 min, 100% B; 3.2 min, 90% B; 6.2 min, 90% B; 6.5 min, 80% B; 10.5 min, 80% B; 10.7 min, 70% B; 13.5 min, 70% B; 13.7 min, 45% B; 16 min, 45% B; 16.5 min, 100% B and 22 min, 100% B. The flow rate was 300 μl/min. Injection volume was 5 μL and column temperature was 25°C. The autosampler temperature was set to 4°C and the injection volume was 5 μL.

The full scan mass spectrometry analysis was performed on a Thermo Q Exactive PLUS with a HESI source which was set to a spray voltage of -2.7kV under negative mode and 3.5kV under positive mode. The sheath, auxiliary, and sweep gas flow rates of 40, 10, and 2 (arbitrary unit) respectively. The capillary temperature was set to 300°C and aux gas heater was 360°C. The S-lens RF level was 45. The m/z scan range was set to 72 to 1000m/z under both positive and negative ionization mode. The AGC target was set to 3e6 and the maximum IT was 200ms. The resolution was set to 70,000. The full scan data was processed with a targeted data pipeline using MAVEN software package [53]. The compound identification was assessed using accurate mass and retention time match to the metabolite standards from the in-house library.

### Mouse studies

In the pneumonia model mice were intranasally infected with 2–4 x 10^7^ cfu of *S*. *aureus* in 50 μl of PBS under anesthesia (ketamine and xylazine). Bacterial loads were enumerated at 24 h post infection from bronchoalveolar lavage fluid (BALF) by washing the airway 3 times with 1 ml of PBS and homogenized lung tissue. In the sepsis model, mice were intraperitoneally infected with 2–4 x 10^7^ cfu of *S*. *aureus* in 100 μl PBS. Mice were infected for 24 h before peritoneal lavage was collected by washing with 3 ml of PBS; lungs and spleens were homogenized to enumerate bacterial counts. In the skin infection model, mice were subcutaneously infected with 2–4 x 10^6^ cfu in 100 μl of PBS under anesthesia. The size of dermonecrosis was measured for 5 days and number of bacteria were counted from homogenized skin tissue at 5 days post infection. Bacterial counts were quantified by using CHROMagar *S*. *aureus* plates (BD Biosciences).

### Statistics

All experiments are performed on at least two separate occasions with at least three biological replicates. *In vitro* experiments were assessed using a parametric student t-test. Multiple comparisons are conducted using a one-way ANOVA with Tukey’s multiple comparison test. Animal data were assessed using a nonparametric Mann-Whitney test or multiple groups using a non-parametric ANOVA with a Kruskwal-Wallis multiple comparison test. Statistics were performed using Prism software (GraphPad, La Jolla LA USA).

The numerical data used in all figures are included in S1 Data.

## Supporting information

S1 Fig Growth of WT and *pgl* strains under different carbon, sulfur and phosphorus sources. WT, *pgl* and *pgl*_c_ strains were grown using Biolog phenotype array plates for A) carbon sources and B) sulfur and phosphorus sources. n = 3 from independent experiments. C) Strains were grown in CDM with the labelled carbon source. n = 8. Graphs show means with standard deviations. ****P<0.0001, **P<0.01, *<P.0.05.
(TIF)

S2 Fig Inactivation of glucose and gluconate import does not impact virulence in an acute pneumonia model of *S*. *aureus* infection. Strains of *S*. *aureus* were inoculated intranasally into mice before euthanasia 24 h later to enumerate bacteria. Each point represents a mouse. Lines display median. **P<0.01.
(TIF)

S3 Fig *S. aureus pgl* shows a defect in biofilm formation in M9 media. *S. aureus* strains were allowed to grow in static M9 cultures for 24 h before A) growth and B) biofilms were quantified. n = 18. Graphs show means with standard deviation. ***P<0.001 and **P<0.01.
(TIF)

S4 Fig Oxidative stress genes are not altered in *pgl* during hydrogen peroxide incubation. *S*. *aureus* strains were incubated in hydrogen peroxide for 4 h. A) Bacterial counts after incubation. n = 10. B) qRT-PCR of genes related to oxidative stress after 2 h incubation in hydrogen peroxide. *katA*, *ahpC*, *sodA*, *bsaA*, n = 7, all other genes n = 4. Graphs show means with standard deviation. ***P<0.0001 and **P<0.01.
(TIF)

S1 Table Carbon source utilization by *S*. *aureus* strains. (XLSX)

S2 Table Growth in different phosphorus and sulfur sources by *S*. *aureus* strains. (XLSX)

S3 Table Differentially expressed genes in *pgl* vs WT and complemented strains. (DOCX)

S4 Table Metabolomics normalized ion counts. (XLSX)

S5 Table Metabolomics normalized ion counts in M9. (XLSX)

S6 Table ^13^C labelling metabolomics. (XLSX)

S1 Data Original data for all graphs presented in manuscript. (XLSX)
