# Supplementary figures and images for: Impact of the pentose phosphate pathway on metabolism and pathogenesis of Staphylococcus aureus
Source: PLoS Pathog. 2023 Jul 13;19(7):e1011531. doi: 10.1371/journal.ppat.1011531 (PMC10368262; doi:10.1371/journal.ppat.1011531)

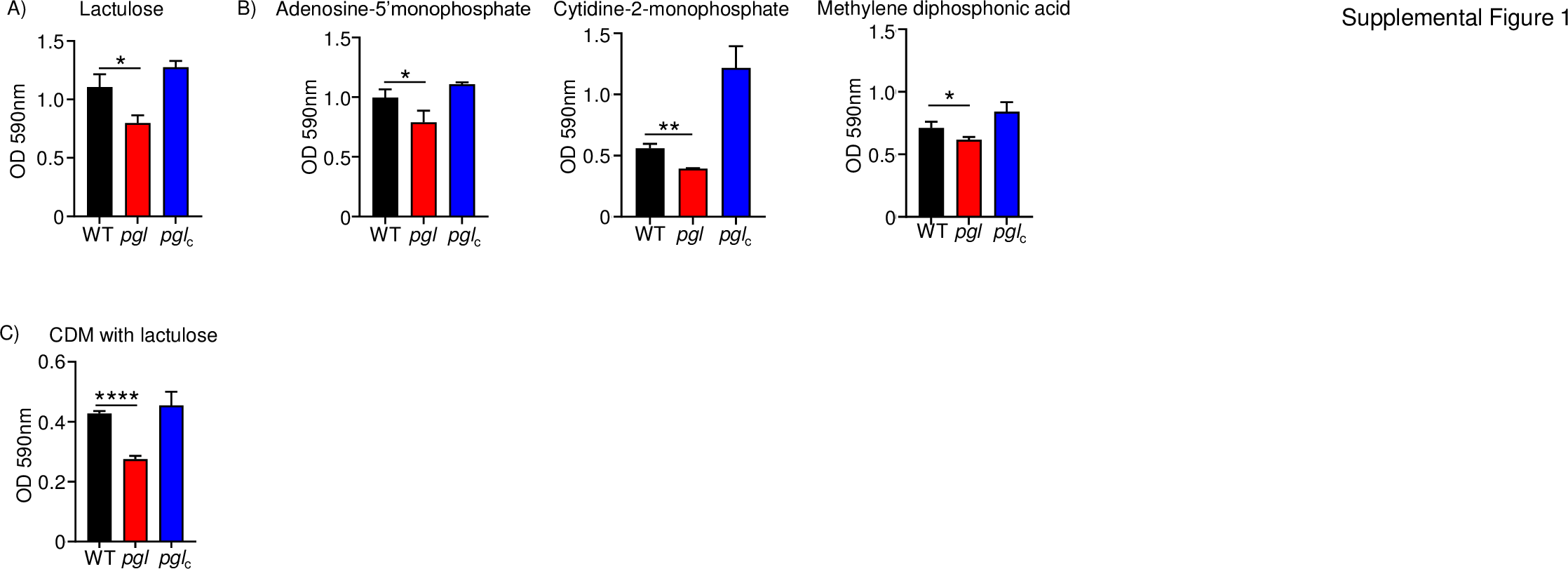

Supplement: S1 Fig — WT, pgl and pglc strains were grown using Biolog phenotype array plates for A) carbon sources and B) sulfur and phosphorus sources. n = 3 from independent experiments. C) Strains were grown in CDM with the labelled carbon source. n = 8. Graphs show means with standard deviations. ****P<0.0001, **P<0.01, *<P.0.05. (TIF) [file ppat.1011531.s001.tif]

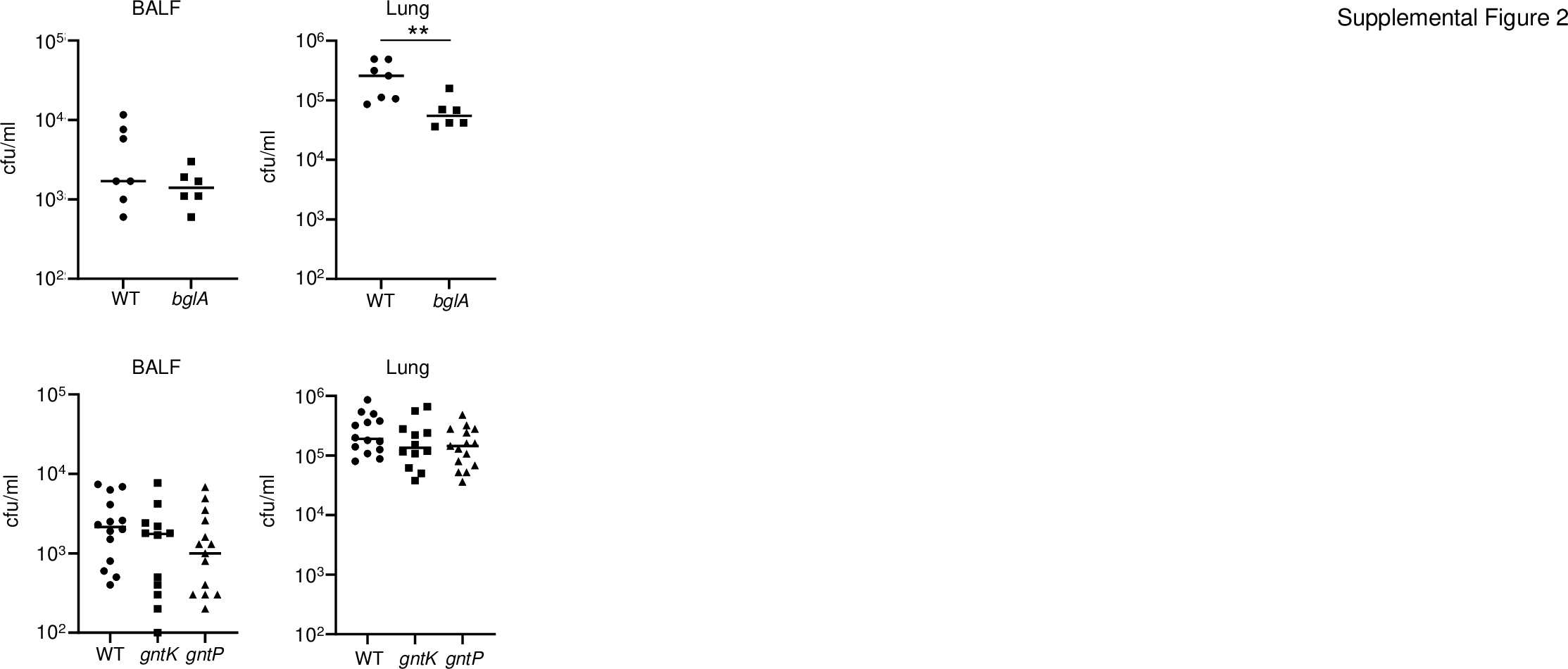

Supplement: S2 Fig — Strains of S. aureus were inoculated intranasally into mice before euthanasia 24 h later to enumerate bacteria. Each point represents a mouse. Lines display median. **P<0.01. (TIF) [file ppat.1011531.s002.tif]

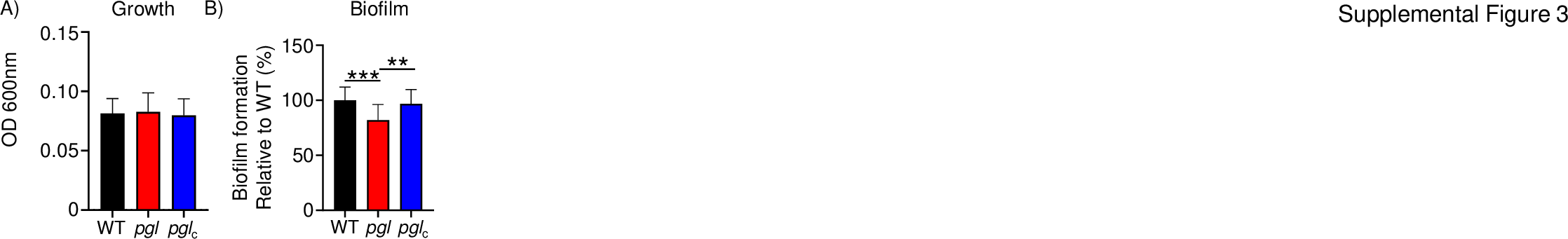

Supplement: S3 Fig — S. aureus strains were allowed to grow in static M9 cultures for 24 h before A) growth and B) biofilms were quantified. n = 18. Graphs show means with standard deviation. ***P<0.001 and **P<0.01. (TIF) [file ppat.1011531.s003.tif]

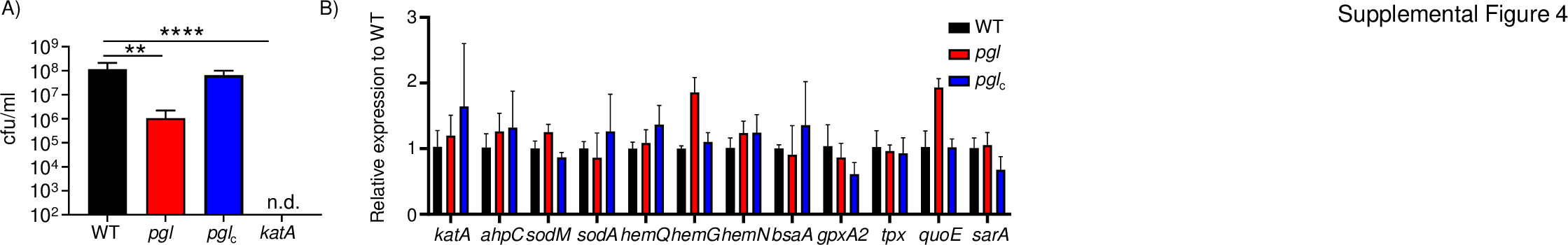

Supplement: S4 Fig — S. aureus strains were incubated in hydrogen peroxide for 4 h. A) Bacterial counts after incubation. n = 10. B) qRT-PCR of genes related to oxidative stress after 2 h incubation in hydrogen peroxide. katA, ahpC, sodA, bsaA, n = 7, all other genes n = 4. Graphs show means with standard deviation. ***P<0.0001 and **P<0.01. (TIF) [file ppat.1011531.s004.tif]
