# Supplementary material for: Impact of the pentose phosphate pathway on metabolism and pathogenesis of Staphylococcus aureus
Source: PLoS Pathog. 2023 Jul 13;19(7):e1011531. doi: 10.1371/journal.ppat.1011531 (PMC10368262; doi:10.1371/journal.ppat.1011531)
Supplement: S3 Table — (DOCX) [file ppat.1011531.s007.docx]

| **Gene** | **Gene ID-putative function** | **WT** | ***pgl*** | ***pgl-comp*** |
| --- | --- | --- | --- | --- |
|  | SAUSA300_0259 - PTS system, IIA component | 1.00 | 32.6 | 0.92 |
| *bglA* | SAUSA300_0260 - 6-phospho-beta-glucosidase | 1.00 | 13.91 | 0.71 |
| *bglR* | SAUSA300_0258 - transcriptional regulator, GntR family | 1.00 | 7.10 | 0.87 |
| *scrA* | SAUSA300_2324 - protein=PTS system, sucrose-specific IIBC component | 1.00 | 15.58 | 0.96 |
|  | SAUSA300_0261 - conserved hypothetical protein | 1.00 | 17.37 | 0.83 |
| *gntP* | SAUSA300_2442 - gluconate permease | 1.00 | 3.32 | 1.10 |
| *pdxT* | SAUSA300_0505 - glutamine amidotransferase | 1.00 | 3.68 | 1.25 |
| *pdxS* | SAUSA300_0504 - pyridoxine biosynthesis protein | 1.00 | 4.54 | 1.21 |
| *gntR* | SAUSA300_2444 - gluconate operon transcriptional repressor | 1.00 | 3.00 | 1.10 |
| *gntK* | SAUSA300_2443 - gluconate kinase | 1.00 | 3.11 | 0.85 |
| *ptsG* | SAUSA300_0191 - PTS system, glucose-specific IIBC component | 1.00 | 3.80 | 1.18 |
| *glcB* | SAUSA300_2476 - phosphotransferase system | 1.00 | 2.99 | 1.14 |
| *sstC* | SAUSA300_0720 - putative iron compound ABC transporter | 1.00 | 2.04 | 1.03 |
|  | SAUSA300_1099 - conserved hypothetical protein | 1.00 | 0.53 | 1.30 |
| *sstB* | SAUSA300_0719 - iron compound ABC transporter, permease protein | 1.00 | 1.52 | 0.89 |
| *deoD* | SAUSA300_0138 - purine nucleoside phosphorylase | 1.00 | 0.39 | 0.66 |
| *pyrR* | SAUSA300_1091 - PyrR bifunctional protein | 1.00 | 0.72 | 1.01 |
| *tet38* | SAUSA300_0139 - putative tetracycline resistance protein | 1.00 | 0.31 | 0.76 |
| *pyrD* | SAUSA300_2526 - dihydroorotate dehydrogenase | 1.06 | 0.47 | 1.04 |
| *sstA* | SAUSA300_0718 - iron compound ABC transporter, permease | 1.00 | 0.87 | 1.00 |
| *pgl* | SAUSA300_1902 - conserved hypothetical protein | 1.00 | 0.30 | 1.78 |
| *pyrP* | SAUSA300_1092 - uracil permease | 1.00 | 0.62 | 0.84 |
| *pyrB* | SAUSA300_1093 - aspartate carbamoyltransferase | 1.00 | 0.35 | 1.12 |
| *pyrC* | SAUSA300_1094 - dihydroorotase | 1.00 | 0.44 | 1.06 |
| *carB* | SAUSA300_1096 - carbamoyl-phosphate synthase | 1.00 | 0.50 | 1.05 |
| *carA* | SAUSA300_1095 - carbamoyl-phosphate synthase | 0.99 | 0.60 | 1.57 |
| *pyrF* | SAUSA300_1097 - orotidine 5'-phosphate decarboxylase | 1.00 | 0.38 | 1.00 |
| *pyrE* | SAUSA300_1098 - orotate phosphoribosyltransferase | 1.00 | 0.44 | 1.17 |

**Table S3. Differentially expressed genes in *pgl* vs WT and complemented strains.**
